# Low-frequency rTMS modulates small-world network properties in an AVH-related brain network in schizophrenia

**DOI:** 10.3389/fpsyt.2025.1578072

**Published:** 2025-04-15

**Authors:** Lin Zhang, Li Guo, Xiaohui Liu, Jing Han, Yuanqiang Zhu, Chaozong Ma, Ye Li, Weiliang Ye

**Affiliations:** ^1^ Tenth Outpatient Department, 986th Hospital, Xijing Hospital, Air Force Medical University, Xi’an, China; ^2^ Department of Psychology, 986th Hospital, Xijing Hospital, Air Force Medical University, Xi’an, China; ^3^ Department of Radiology, Xijing Hospital, Air Force Medical University, Xi’an, China; ^4^ Military Medical Psychology School, Air Force Medical University, Xi’an, China; ^5^ No. 968 Hospital of Chinese People’s Liberation Army, Jinzhou, China; ^6^ Department of Pharmaceutics, School of Pharmacy, Air Force Military Medical University, Xi’an, China

**Keywords:** schizophrenia, auditory, verbal hallucinations, repetitive transcranial magnetic stimulation (rTMS), small-world network, temporoparietal junction

## Abstract

**Background:**

Auditory verbal hallucinations (AVH) are a core symptom of schizophrenia, often persisting despite pharmacological treatment. Repetitive transcranial magnetic stimulation (rTMS), particularly low-frequency rTMS targeting the left temporoparietal junction (TPJ), has shown promise in alleviating AVH symptoms by modulating dysfunctional brain connectivity. However, the network-level effects of rTMS remain incompletely understood, particularly in terms of small-world network properties, which provide insights into local and global network efficiency. Furthermore, most previous studies have analyzed whole-brain networks, lacking specificity regarding disease-relevant circuits.

**Objective:**

This study aimed to investigate how low-frequency rTMS modulates the small-world network properties of a refined AVH-related network composed of 35 brain regions specifically implicated in hallucination generation and rTMS treatment effects, thereby providing a more targeted perspective on network reorganization. Healthy controls (HCs) were included as a reference to determine whether rTMS normalizes network alterations in schizophrenia. Additionally, a responder vs. non-responder analysis was conducted to assess individual variability in treatment response.

**Methods:**

A total of 50 schizophrenia patients with persistent AVH underwent 15 sessions of 1 Hz rTMS over the left TPJ. Resting-state fMRI data were collected before and after treatment to assess functional connectivity within the predefined 35-region AVH-related network. small-worldness (σ), normalized clustering coefficient (γ), and normalized characteristic path length (λ), as well as functional segregation (clustering coefficient [Cp], local efficiency [El]) and functional integration (global efficiency [Eg], characteristic path length [Lp])—were analyzed before and after rTMS. Clinical symptom severity was assessed using the Auditory Hallucination Rating Scale (AHRS).

**Results:**

At baseline, schizophrenia patients exhibited disrupted small-world properties, with significantly lower σ, Cp, El, and Eg compared to healthy controls, reflecting impaired network organization, reduced local clustering, and inefficient global communication. Following rTMS, small-worldness (σ), local efficiency (El), and global efficiency (Eg) showed significant improvement, suggesting partial restoration of network efficiency. Functional connectivity analyses revealed significant reductions in hyperconnectivity between the right middle temporal gyrus (MTG) and superior putamen, as well as between the left TPJ and left lateral prefrontal cortex (LPFC). Notably, responders showed greater connectivity changes, which were correlated with AVH severity reduction, highlighting the role of network modulation in treatment response.

**Conclusion:**

These findings support the network dysregulation model of AVH in schizophrenia and demonstrate that rTMS can modulate AVH-related connectivity, partially restoring network efficiency. The inclusion of HCs provides evidence that rTMS-induced changes align with normative network patterns, and the responder analysis suggests that connectivity modulation is linked to clinical improvement. This study offers new insights into the neurobiological mechanisms of rTMS treatment and underscores the need for biomarker-driven, individualized neuromodulation strategies for schizophrenia.

## Introduction

Auditory verbal hallucinations (AVH) are a hallmark symptom of schizophrenia (1), significantly impairing patients’ quality of life and often persisting despite pharmacological treatment (2). Repetitive transcranial magnetic stimulation (rTMS), particularly low-frequency (1 Hz) stimulation over the left temporoparietal junction (TPJ), has been extensively studied as a non-invasive intervention for AVH (3, 4). Randomized controlled trials (RCTs) and meta-analyses have demonstrated its clinical efficacy. A systematic review of 41 RCTs (N=1473) found significant reductions in AVH severity and PANSS-positive scores, though variability in study quality has led to inconsistent clinical recommendations (5). Another meta-analysis reported a large effect size (1.04, p=0.002) for AVH reduction in sham-controlled trials (6). Additional reviews support 1 Hz rTMS over the TPJ as the most effective protocol for AVH (7, 8). Previous research, including our own work, has demonstrated that low-frequency rTMS targeting the TPJ can induce functional and structural changes in brain connectivity, leading to clinical improvements in AVH (9–12). The proposed mechanism involves reducing cortical hyperexcitability in the TPJ and modulating abnormal connectivity with auditory and prefrontal networks, thereby improving self-monitoring and speech processing. However, the precise neural mechanisms underlying these effects remain incompletely understood, necessitating further investigation (7).

Schizophrenia is increasingly recognized as a “disconnection syndrome,” characterized by widespread disruptions in connectivity within and between brain networks (13). The therapeutic effects of low-frequency rTMS have been linked to its ability to modulate brain connectivity across multiple dimensions (14). Changes in connectivity have been observed not only in the TPJ itself but also in an extended network of regions involved in auditory processing, language function, and self-monitoring, including the dorsolateral prefrontal cortex (DLPFC), anterior cingulate cortex (ACC), insula, and supplementary motor area (SMA) (9, 15). These regions, which are frequently disrupted in schizophrenia, may collectively contribute to the observed clinical improvements following rTMS treatment. Evidence suggests that this modulation may influence not only the synchronization of activity between brain regions but also the integrity of the pathways that support these interactions (10). Furthermore, the interplay between dynamic activity and underlying structural frameworks appears to become more aligned, reflecting improved network organization (11). Adjustments in the directional influence of key brain areas also highlight the potential for restoring balance within disrupted circuits. Together, these findings suggest that low-frequency rTMS holds promise in addressing the complex connectivity deficits characteristic of schizophrenia, thereby contributing to the alleviation of AVH symptoms.

In recent years, the concept of small-world networks has emerged as a powerful framework for understanding the brain’s functional organization (16, 17). Small-world networks, characterized by a balance between high local clustering and short global path lengths, offer an efficient structure for both specialized processing and global information integration. This framework goes beyond simple connectivity metrics by capturing the broader topological properties of neural networks, providing a more comprehensive understanding of how brain regions interact. Compared to traditional approaches that focus solely on functional or structural connections, small-world analysis allows for a deeper exploration of network efficiency and resilience, making it particularly valuable in studying complex brain disorders such as schizophrenia (18, 19).

Much of the existing research utilizing small-world network analysis has been conducted at the whole-brain level (20). While informative, these studies often lack specificity, as they may overlook the unique contributions of disease-specific or treatment-relevant networks. This generalized approach limits the ability to identify precise network characteristics associated with auditory verbal hallucinations (AVH) or their modulation by therapeutic interventions such as low-frequency rTMS. Previous research has identified a 25-region AVH-related network based on data-driven connectivity analyses in schizophrenia (21). Building on this foundation, we incorporated 10 additional regions identified from our prior studies, which are functionally linked to both AVH generation and rTMS effects (9–11, 21, 22). This refined network includes key regions such as the left superior temporal gyrus (STG), left inferior parietal lobule (IPL), right superior frontal gyrus (SFG), posterior cingulate cortex (PCC), and right putamen, as well as regions implicated in executive and salience processing, including the dorsolateral prefrontal cortex (DLPFC), anterior cingulate cortex (ACC), insula, and supplementary motor area (SMA). By focusing on this well-defined AVH-related network, we enhance sensitivity to detecting rTMS-induced connectivity changes while avoiding the statistical limitations of whole-brain approaches, which may introduce irrelevant regions. This targeted approach enables us to examine small-world network properties and assess how rTMS influences network efficiency and functional reorganization, providing novel insights into the neurobiological mechanisms underlying AVH symptom alleviation and treatment response.

The focus of this research is to examine how the small-world network properties of a targeted AVH-related network, composed of 35 brain regions, are altered following low-frequency rTMS treatment. We hypothesize that rTMS may lead to significant improvements in the small-world characteristics of this network, enhancing both local clustering and global efficiency, which are critical for effective neural communication. These changes may contribute to the reduction of hallucination severity and support recovery in cognitive and emotional processing in individuals with schizophrenia. By specifically analyzing the small-world properties of this refined network, we aim to overcome the limitations of whole-brain studies and provide a more nuanced understanding of how rTMS modulates disease-specific connectivity patterns. To further investigate the network-level mechanisms underlying rTMS efficacy, we included a healthy control (HC) group to determine whether functional connectivity and network properties in schizophrenia patients normalize following rTMS treatment. This comparison provides a reference point for evaluating connectivity disruptions in AVH and their modulation by rTMS. Additionally, recognizing that individual treatment response varies, we conducted a responder vs. non-responder analysis to examine whether specific changes in network properties correlate with clinical improvements in AVH severity. The primary aim of this study is to determine whether rTMS modulates aberrant functional connectivity associated with AVH in schizophrenia, providing insight into the neural mechanisms underlying hallucinatory experiences and their potential reversibility through neuromodulation.

## Materials and methods

### Patients

A total of 50 right-handed patients diagnosed with schizophrenia and experiencing persistent auditory verbal hallucinations (AVH) were recruited from the Department of Psychiatry at Xijing Hospital. Additionally, 50 sex-, age-, and handedness-matched healthy controls (HC) were recruited via advertisements from the local community. All participants were aged between 18 and 45 years, and diagnoses of schizophrenia were confirmed by experienced psychiatrists using the Diagnostic and Statistical Manual of Mental Disorders, Fifth Edition (DSM-5) criteria. The inclusion criteria for patients required a confirmed diagnosis of schizophrenia based on DSM-5 criteria, the presence of persistent AVH occurring daily despite treatment with at least two antipsychotic medications, and at least five times per day over the past month despite treatment (23). To ensure stability, only patients who had been on a consistent dose of antipsychotic medication for at least 12 months were included. All schizophrenia patients were receiving stable doses of antipsychotic medications throughout the study period. Medication dosage was recorded in chlorpromazine equivalents (CPED, mg/day) to allow standardized comparisons, following previous studies in our research group (10, 11). Exclusion criteria encompassed a history of neurological disorders, significant head injury, or other major medical conditions, as well as substance abuse, a positive urine toxicology screen, contraindications to MRI (e.g., metallic implants or severe claustrophobia), pregnancy, or nursing.

For the HC group, inclusion required no lifetime history of psychotic, mood, or substance use disorders, as confirmed by the Structured Clinical Interview for DSM-IV Non-Patient version (SCID-NP), and no first-degree family history of psychiatric disorders. Exclusion criteria for both groups included significant medical or neurological illnesses, current misuse of substances other than nicotine, and any MRI contraindications. All participants provided written informed consent prior to participation. The study was approved by the Research Ethics Committee of Xijing Hospital, Fourth Military Medical University (Approval Number: XJYYLL-2021065) and was conducted in accordance with the Declaration of Helsinki (1975, revised 2008).

### Clinical and neurocognitive assessments

Schizophrenia symptoms were assessed using the Positive and Negative Syndrome Scale (PANSS), which evaluates positive, negative, and general psychopathology symptoms (24). The severity of auditory verbal hallucinations (AVH) was further quantified using the Auditory Verbal Hallucinations Rating Scale (AHRS), which measures the frequency, duration, and intensity of AVH (25). All clinical assessments were conducted by experienced psychiatrists within three days before and after the intervention to ensure consistency and reliability.

### rTMS procedures

Repetitive transcranial magnetic stimulation (rTMS) was applied to the left temporoparietal junction (TPJ), a key region implicated in the pathophysiology of auditory verbal hallucinations (AVH) in schizophrenia (26). Similar with our previous studies (9, 11), Stimulation was delivered using a Magstim Rapid system equipped with a figure-eight coil (YIRUIDE YCD-I, Wuhan, China), with the target site localized based on the 10-20 EEG system (midpoint between T3 and P3 electrodes). The protocol utilized low-frequency stimulation at 1 Hz, with an intensity set at 110% of the individual’s resting motor threshold (RMT). The selection of 1 Hz stimulation was based on its well-established inhibitory effects on cortical excitability, making it particularly suitable for reducing hyperactivity in the left TPJ. Each treatment session consisted of 600 pulses, delivered across 60 trains in a structured pattern of 10 seconds of stimulation followed by a 5-second interval. Sessions lasted 15 minutes per day and were administered for 15 consecutive days. To evaluate the safety and tolerability of rTMS, all participants were monitored for potential side effects throughout the treatment period. Side effects were assessed using a standardized checklist covering headache, scalp discomfort, dizziness, fatigue, and other adverse events, as recommended in previous rTMS studies. Participants were asked to report any discomfort during or after sessions, and all adverse events were documented.

### MRI data acquisition

MRI data were acquired using a 3.0 T Discovery MR750 scanner (GE, USA) with an 8-channel head coil. Foam padding and earplugs were used to reduce head motion and scanner noise. Participants were instructed to remain still, stay awake, and keep their eyes closed during the scans. Resting-state fMRI (rs-fMRI) images were obtained with the following parameters: TR = 2000 ms, TE = 40 ms, slice thickness = 3.5 mm, 45 axial slices, flip angle = 90°, field of view (FOV) = 260 × 260 mm, and matrix size = 64 × 64, with a total of 180 volumes acquired over a 7-minute scan. Structural T1-weighted images were collected using a TR of 9.1 ms, TE of 3.2 ms, flip angle = 10°, FOV = 240 × 240 mm, matrix size = 256 × 256, providing 1 mm isotropic resolution. Healthy controls underwent a single MRI scan, while patients underwent two scans—one before and one after rTMS treatment. Imaging parameters were optimized to enhance blood oxygen level-dependent (BOLD) signal detection and minimize susceptibility artifacts, ensuring suitability for functional connectivity analysis in clinical populations.

### Data preprocessing

Resting-state fMRI data were preprocessed using the Data Processing and Analysis for Brain Imaging (DPABI) toolbox, which integrates functionalities from the Resting-State fMRI Data Analysis Toolkit (REST) and Statistical Parametric Mapping (SPM12). The first 10 volumes were discarded to allow for magnetization equilibrium. Slice timing correction was applied to adjust for temporal differences between slices, followed by realignment to correct for head motion. Participants with head movements exceeding 3 mm of translation or 3° of rotation in any direction were excluded.

Each participant’s T1-weighted structural image was coregistered to their functional images and segmented into gray matter, white matter, and cerebrospinal fluid (CSF). Functional images were normalized to Montreal Neurological Institute (MNI) space using the DARTEL (diffeomorphic anatomical registration through exponentiated Lie algebra) method and smoothed with a 6 mm full-width at half maximum (FWHM) Gaussian kernel. Linear detrending and band-pass filtering (0.01–0.08 Hz) were applied to minimize physiological noise. To further control for confounds, nuisance regression was performed using the Friston-24 head motion parameters, as well as signals from white matter and CSF. Global signal regression was not applied to avoid potential biases in connectivity analysis.

### Network construction and analysis

Brain network properties were analyzed using GRETNA software (27), applying graph theory-based methods to assess functional connectivity within 35 predefined brain regions associated with auditory verbal hallucinations (**Table 1**). Pearson correlation coefficients were computed between the time series of all possible pairs of these 35 regions, generating an individual 35 × 35 correlation matrix for each participant. Following the approach of prior studies (e.g., Song et al., 2020), each correlation matrix was transformed into an undirected binarized network through sparsity thresholding, ranging from 8% to 50% in increments of 0.01, ensuring comparable network density across participants.

**Table 1:** Auditory verbal hallucination (AVH)-related brain regions and their MNI coordinates used in small-world network analysis.

Node Number	Anatomical Location	Abbreviations	MNI Coordinates (X, Y, Z)		
Node 1	Right Temporal pole	Tpole.R	58	3	-24
Node 2	Right Fusiform gyrus/BA19	BA19.R	20	-64	-24
Node 3	Left BA19	BA19.L	-25	-58	-23
Node 4	Left parahippocampus	PHP.L	-35	-26	-22
Node 5	Right orbital frontal cortex	OFC.R	27	36	-21
Node 6	Left Inferior frontal gyrus	IFG.L	-45	19	-21
Node 7	Inferior medial prefrontal cortex	MPFC	2	39	-19
Node 8	Right inferior putaman	IPUT.R	24	7	-19
Node 9	Left BA21	BA21	-62	-26	-18
Node 10	Left primary visual cortex	VIS.L	-17	-66	-17
Node 11	Left putamen	PUT.L	-18	15	-16
Node 12	Right superior putamen	DPUT.R	23	11	-10
Node 13	Right insula/auditory cortex	INS.L	41	-8	-10
Node 14	Right lateral prefrontal cortex	LPFC.R	55	25	5
Node 15	Medial prefrontal cortex	MFC	-8	61	6
Node 16	Left lateral prefrontal cortex	LPFC.L	-52	24	8
Node 17	Left BA19	BA19.L	-30	-85	9
Node 18	Right BA40	BA40.R	60	-39	18
Node 19	Right lateral prefrontal cortex	PFC.R	53	13	19
Node 20	Posterior cingulated cortex	PCC	3	-39	20
Node 21	Left sensory cortex	SEN.L	-59	-21	21
Node 22	Left superior prefrontal cortex	SPFC.L	-51	17	28
Node 23	Anterior cingulate	ACC	11	16	30
Node 24	Left BA39	BA39.L	-53	-45	30
Node 25	Right sensory cortex	SEN.R	42	-29	34
Node 26	Left Temporoparietal Junction	TPJ.L	-50	-40	20
Node 27	Left Superior Temporal Gyrus	STG.L	-60	-30	10
Node 28	Right Middle Temporal Gyrus	MTG.R	50	-50	10
Node 29	Left Inferior Temporal Gyrus	ITG.L	-55	-60	-5
Node 30	Right Superior Frontal Gyrus	SFG.R	20	60	20
Node 31	Left Inferior Parietal Lobule	IPL.L	-45	-50	40
Node 32	Supplementary Motor Area	SMA	10	0	60
Node 33	Posterior Cingulate Cortex	PCC	3	-39	20
Node 34	Right Putamen	PUT.L	24	7	-19
Node 35	Primary Visual Cortex	VIS.R	17	-66	-17

Graph theoretical metrics were calculated to assess functional segregation, integration, and small-world properties of the networks. Functional segregation was evaluated using the clustering coefficient (Cp), normalized clustering coefficient (γ), and local efficiency (Eloc), while functional integration was assessed via characteristic path length (Lp), normalized characteristic path length (λ), and global efficiency (Eglob). Small-worldness (σ = γ/λ) was also computed as an overall network efficiency measure. To facilitate group comparisons, the area under the curve (AUC) for each network metric across the sparsity range was calculated, providing a robust and comprehensive assessment of network topology.

### Statistical analysis

Group differences in demographic characteristics were assessed using chi-square tests for categorical variables and Student’s t-tests for continuous variables, conducted in SPSS (IBM SPSS Statistics for Windows, version 22.0, IBM Corp.). To analyze network changes, two-sample t-tests were applied to compare baseline network metrics between patients and healthy controls (HCs). Paired t-tests were used to evaluate within-group changes in network properties following rTMS intervention.

To control for confounding variables, age, sex, and mean frame-wise displacement (FD) from preprocessing were included as covariates in the statistical models. Multiple comparisons were corrected using the false discovery rate (FDR) criterion (p < 0.05). Additionally, network-based statistics (NBS) were applied to identify significant alterations in functional connectivity patterns before and after treatment. Finally, Pearson correlation analyses were conducted to explore associations between changes in network metrics (AUC values) and clinical symptom improvement, further adjusting for multiple comparisons where necessary.

## Results

### Demographic and clinical characteristics

The demographic and clinical characteristics of schizophrenia (SZ) patients and healthy controls (HC) are summarized in **Table 2**. There were no significant differences between the groups in terms of age, sex distribution, or handedness, with both groups being predominantly right-handed. However, SZ patients had significantly fewer years of education compared to HCs.

**Table 2:** Demographic and clinical characteristics of the HC and SZ.

Characteristic	SZ (n = 47)	HC (n = 48)	Statistical value	*P* value
Age: years	24.5 ± 5.8	24.9 ± 5.0	-0.62^a^	0.53
Gender (male/female)	27/20	29/19	0.02^b^	0.88
Education: years	11.5 ± 2.8	15.3 ± 1.9	-10.72^a^	**<0.001**
Handedness (right/left)	47/0	48/0	<0.01^b^	0.99
Frame-wise displacement	0.28 ± 0.05	0.27 ± 0.03	1.81^a^	0.08
Duration of illness: month	16.4 ± 17.0	NA	NA	NA
AHRS	25.36 ± 5.23	NA	NA	NA
PANSS scores				
Positive score	22.4 ± 4.8	NA	NA	NA
Negative score	18.7 ± 5.4	NA	NA	NA
General score	44.3 ± 7.8	NA	NA	NA
Total score	84.3 ± 11.2	NA	NA	NA

SZ, schizophrenia; HC, healthy controls; AHRS: Auditory Verbal Hallucination Scale; PANSS, the Positive and Negative Syndrome Scale; NA, not applicable.

^a^Value from the independent-samples t test.

^b^Value from the chi-square test.

Bold values indicate a statistically significant difference with a p-value less than 0.05.

### Treatment effects on auditory hallucinations and symptoms

Following rTMS treatment, schizophrenia patients exhibited a significant reduction in auditory verbal hallucinations (AVH), as reflected in the AHRS scores, which decreased from 25.2 ± 5.1 before treatment to 13.1 ± 3.4 after treatment (t = -12.76, p < 0.001). This indicates a marked decrease in the frequency, duration, and intensity of hallucinations. The PANSS scores showed significant reductions in positive and total symptom scores, reflecting a notable improvement in overall symptom severity. However, there were no significant changes in the negative and general symptom scores, indicating that rTMS primarily targeted positive symptoms. Detailed results are presented in **Table 3**. Besides, no severe adverse effects were observed, and any mild side effects (e.g., transient headache or scalp discomfort) resolved without medical intervention.

**Table 3:** Clinical characteristics of 40 patients before and after treatment.

Characteristic	Before medication	After medication	Statistical value	*P* value
AHRS	25.2 ± 5.1	13.1 ± 3.4	-12.76	**<0.001**
PANSS scores				
Positive score	21.5 ± 4.1	18.3 ± 3.0	-4.63	**<0.001**
Negative score	19.2 ± 4.7	19.6 ± 5.7	0.35	0.72
General score	43.0 ± 8.6	44.3 ± 8.1	0.69	0.49
Total score	89.7 ± 11.6	79.3 ± 11.6	-3.71	**<0.001**

AHRS, Auditory Verbal Hallucination Scale; PANSS, the Positive and Negative Syndrome Scale.Bold values indicate a statistically significant difference with a p-value less than 0.05.

### Changes in small-world network properties

Across the sparsity range of 0.08–0.48, the functional brain networks of both schizophrenia (SZ) patients and healthy controls (HCs) exhibited characteristic small-world properties (σ > 1, λ > 1, γ > 1). However, differences were observed in small-world metrics (σ, λ, γ), functional segregation (El, Cp), and functional integration (Eg, Lp) across groups, indicating altered network organization in schizophrenia.

#### Small-world properties (σ, λ and γ)

**Figure 1:**
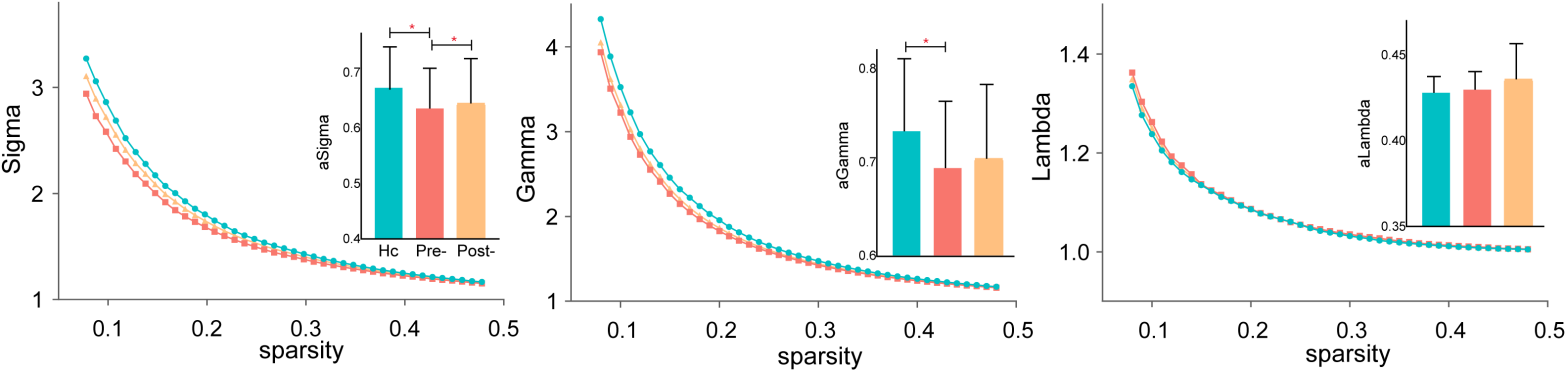
Small-world properties in schizophrenia before and after rTMS treatment. Significant differences in small-worldness (σ) and characteristic path length (λ) were observed between pre-treatment SZ patients and HCs, while normalized clustering coefficient (γ) remained similar across groups. *P < 0.05.

As shown in **Figure 1**, before treatment, σ significantly differed across all three groups (HCs, pre-treatment SZ, and post-treatment SZ), indicating overall disruptions in network efficiency in SZ patients. The characteristic path length (λ) was significantly different between HCs and pre-treatment SZ patients, suggesting reduced global network integration in the patient group. However, γ did not show significant differences among the three groups, indicating that the relative balance of local clustering remained stable despite disease-related network alterations.

#### Functional segregation (El and Cp)

**Figure 2:**
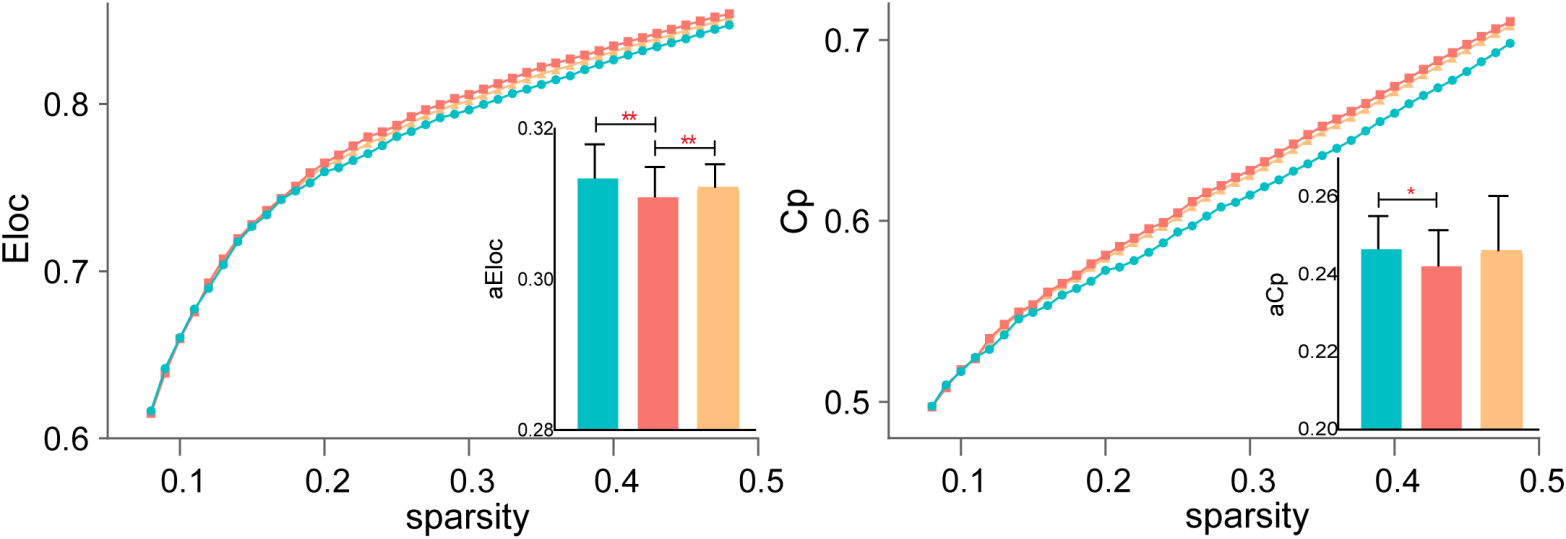
Functional segregation changes following rTMS treatment. Local efficiency (El) significantly increased after rTMS, suggesting partial restoration of local network communication, while clustering coefficient (Cp) remained significantly reduced in SZ patients compared to HCs. *P < 0.05, **P < 0.01.

Before treatment, SZ patients exhibited lower Cp compared to HCs, reflecting reduced local clustering and impaired functional segregation. Additionally, El showed significant differences across all three groups, suggesting widespread abnormalities in local efficiency and weakened local network communication in SZ patients. After rTMS treatment, El significantly increased, indicating a partial restoration of local efficiency, while Cp remained significantly different from HCs, suggesting persistent deficits in local clustering despite treatment. Results are shown in **Figure 2**.

#### Functional integration (Eg and Lp)

**Figure 3:**
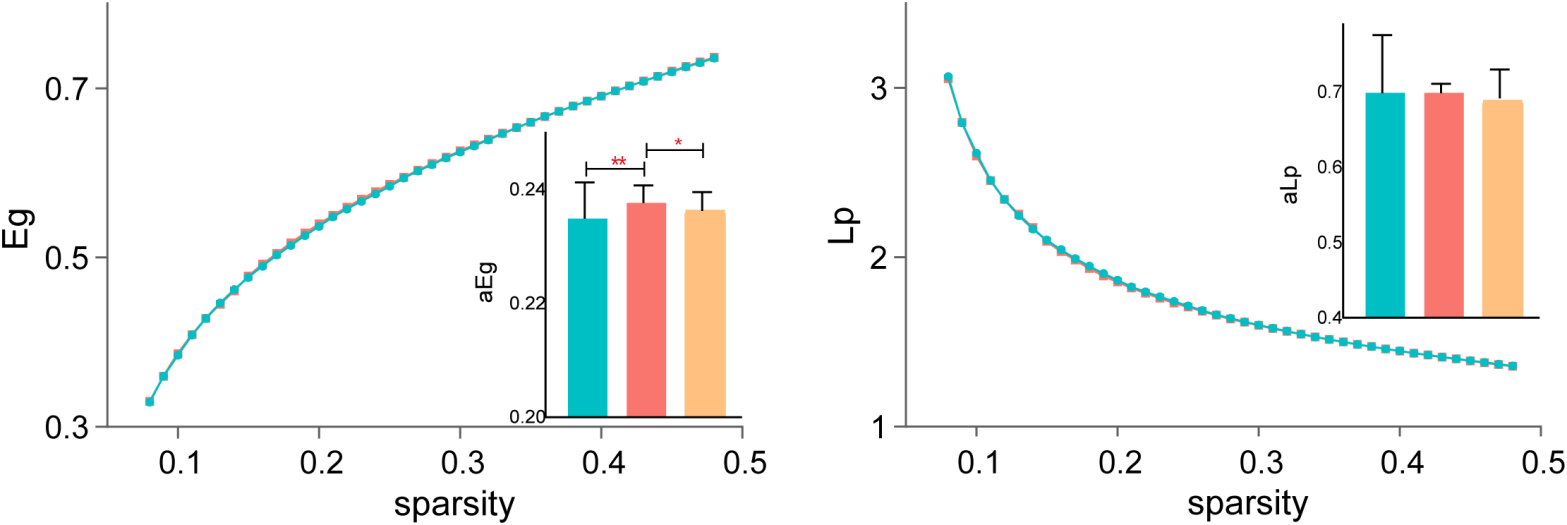
Changes in functional integration after rTMS. Global efficiency (Eg) showed significant improvement post-treatment, while characteristic path length (Lp) remained stable across all groups. *P < 0.05, **P < 0.01.

Both Eg and Lp are key indicators of network integration, reflecting the brain’s ability to efficiently transfer information between distant regions. Eg showed significant differences across all three groups, indicating global efficiency impairments in SZ patients before treatment and partial recovery following rTMS. However, Lp did not significantly differ across groups, suggesting that characteristic path length remained stable despite changes in efficiency metrics. **Figure 3** displays the detailed results.

### Connectivity and correlation results

**Figure 4:**
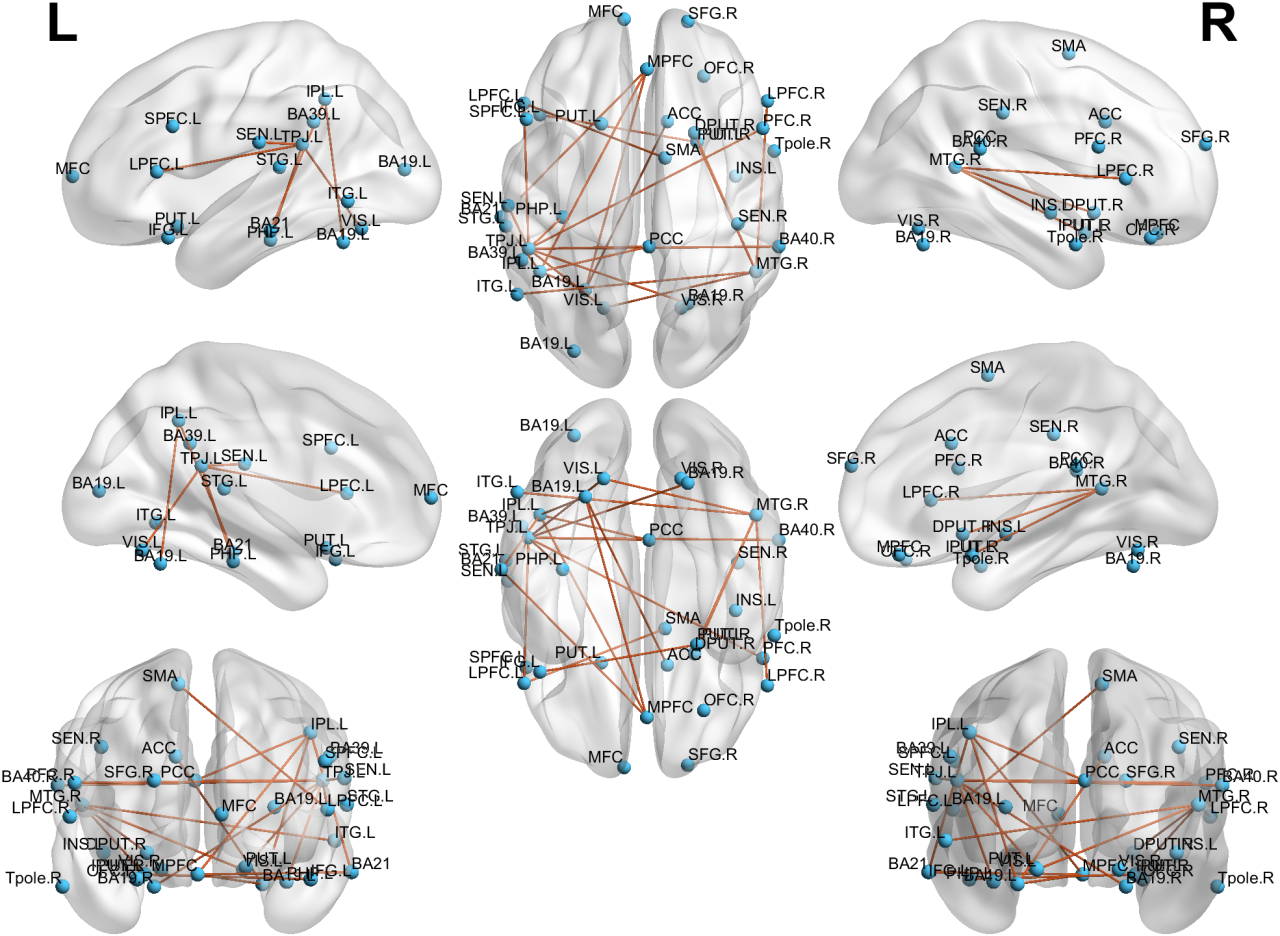
Alterations in connectivity post-rTMS treatment. Significant reductions in connectivity were observed between key regions, including the MTG, TPJ, putamen, and various areas of the PFC, suggesting widespread network reorganization.

After applying network-based statistics (NBS) correction (p < 0.05), schizophrenia patients exhibited altered functional connectivity following rTMS treatment, with significant reductions in key network connections. The primary affected regions included the middle temporal gyrus (MTG), temporoparietal junction (TPJ), and putamen (inferior and superior), as well as multiple subdivisions of the prefrontal cortex (PFC), including inferior, medial, lateral, and superior PFC, alongside the inferior parietal lobule (IPL) and Brodmann area 19 (BA19). These findings indicate widespread network reorganization post-treatment, particularly within circuits associated with auditory processing, language integration, and cognitive control. Detailed connectivity changes are illustrated in **Figure 4**.

**Figure 5:**
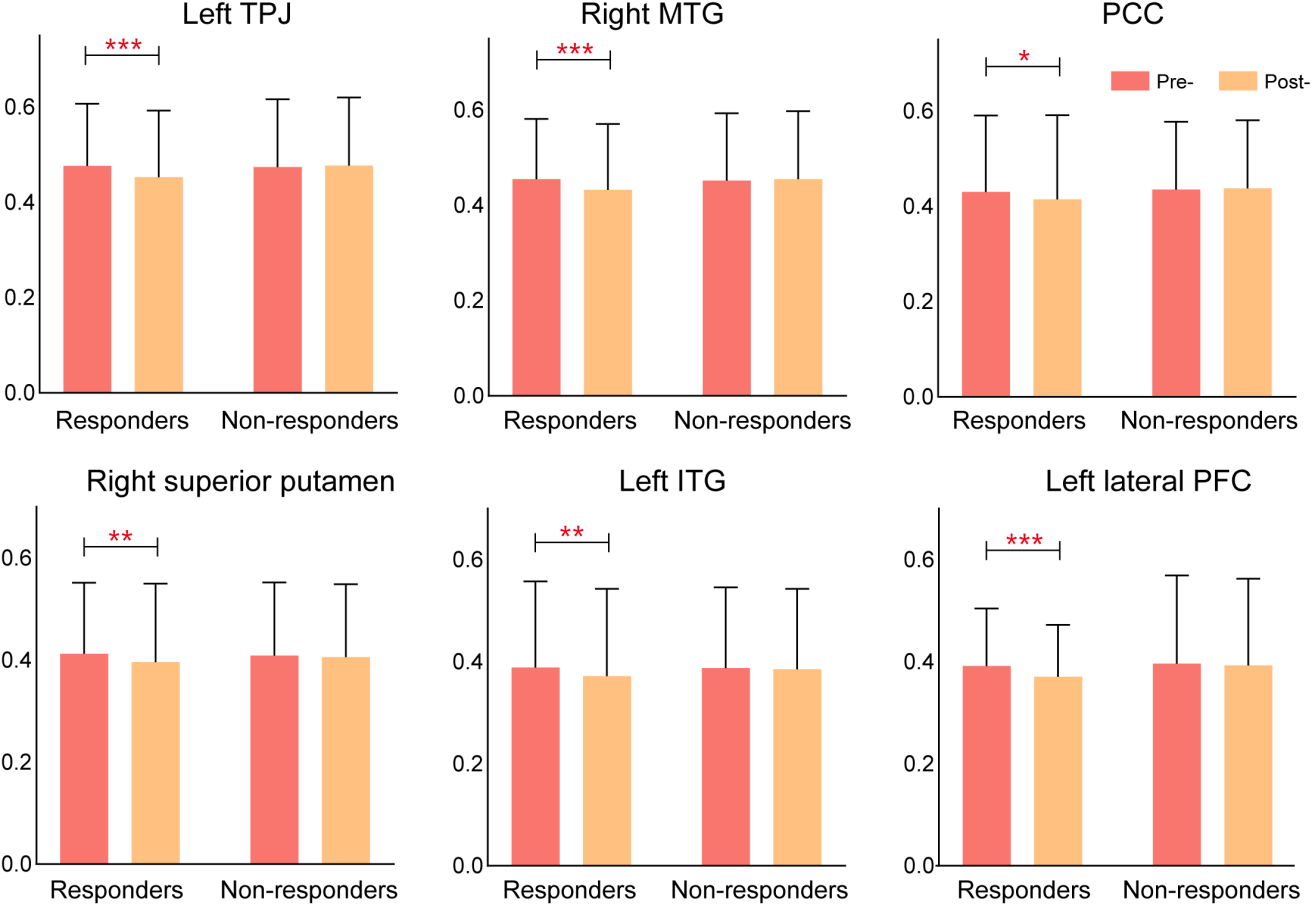
Correlation between connectivity changes and AVH symptom improvement. Reduced connectivity between the left TPJ-left lateral PFC and right MTG-right superior putamen was significantly correlated with reductions in AHRS scores, reflecting the impact of connectivity changes on AVH. *P < 0.05, **P < 0.01, ***P < 0.005.

Moreover, a significant association was found between AHRS score reductions and changes in functional connectivity. Specifically, decreased connectivity between the left TPJ and left lateral PFC (*r*= 0.32, *p* = 0.04), as well as between the right MTG and right superior putamen (*r*= 0.45, *p* = 0.004), was significantly correlated with improvements in AVH symptoms. This suggests that modulation of these pathways may be a key mechanism underlying the therapeutic effects of rTMS. The correlation results are presented in **Figure 5**.

To further investigate individual variability in treatment response, schizophrenia patients were categorized into responders and non-responders based on their improvement in auditory verbal hallucinations (AVH). Responders were defined as patients who exhibited a ≥50% reduction in AHRS scores from baseline to post-treatment, whereas non-responders showed less than 50% improvement. Based on this classification, 67.5% of patients (27/40) were identified as responders, while 32.5% (13/40) were classified as non-responders. No significant differences in CPED doses were found between groups (p > 0.05). This approach ensures that the observed effects of rTMS are not confounded by medication variability.

**Figure 6:**
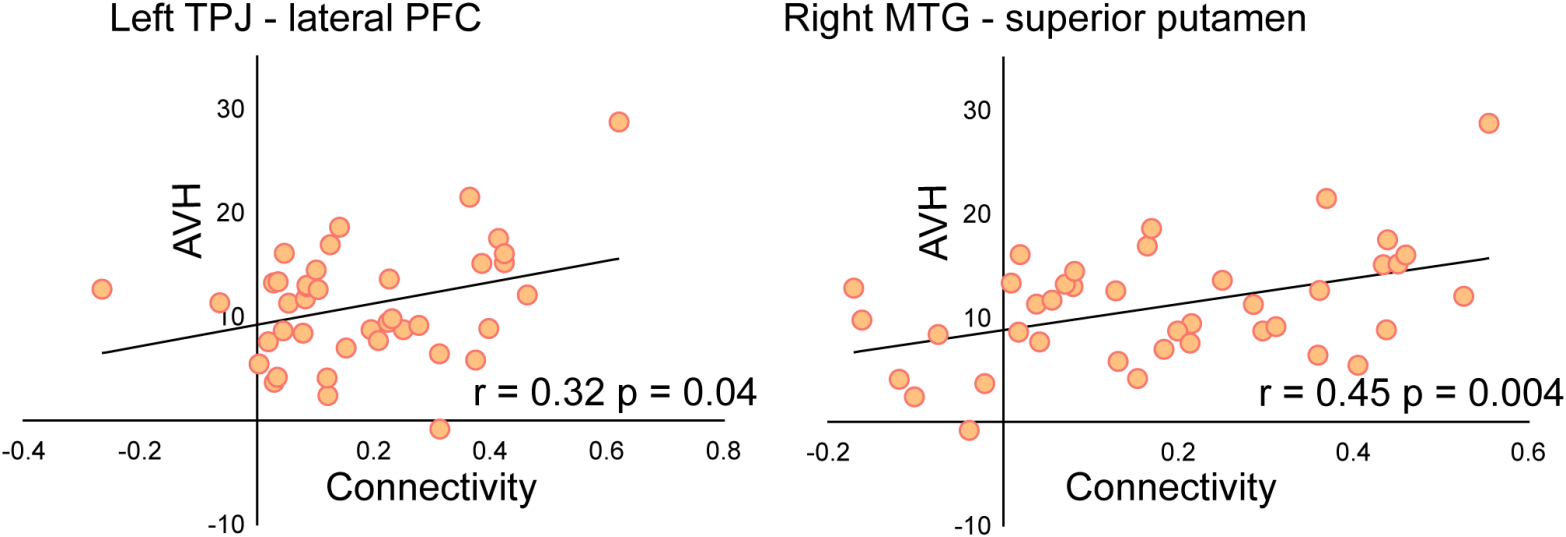
Connectivity changes in rTMS responders vs. non-responders. Responders exhibited significant reductions in connectivity across multiple brain regions, while non-responders showed minimal changes, suggesting that connectivity modulation is associated with treatment response.

Comparative analyses revealed marked differences in functional connectivity changes between responders and non-responders following rTMS treatment. Responders exhibited significant reductions in connectivity across multiple key regions, particularly in the left temporoparietal junction (TPJ), right middle temporal gyrus (MTG), posterior cingulate cortex (PCC), right superior putamen, left inferior temporal gyrus(ITG), and the left lateral prefrontal cortex (LPFC), suggesting a reorganization of brain networks involved in AVH processing. In contrast, non-responders showed minimal or no significant connectivity changes, indicating that the absence of network modulation may underlie their lack of clinical improvement. These findings suggest that rTMS exerts its therapeutic effects by selectively altering functional connectivity in key cortical and subcortical networks, and that the extent of connectivity modulation may serve as a potential biomarker for predicting treatment response in schizophrenia patients with AVH (**Figure 6**).

## Discussion

The present study demonstrated that schizophrenia patients with auditory verbal hallucinations exhibit altered small-world network properties and disrupted functional connectivity, which were partially restored following low-frequency rTMS treatment. Specifically, at baseline, schizophrenia patients showed lower small-worldness (σ), clustering coefficient (Cp), local efficiency (El), and global efficiency (Eg) compared to healthy controls, indicating disrupted network topology, weakened local clustering, and impaired brain-wide communication. After 15 sessions of 1 Hz rTMS targeting the left temporoparietal junction (TPJ), small-worldness (σ), local efficiency (El), and global efficiency (Eg) showed significant increases, suggesting a partial restoration of network efficiency. In addition, functional connectivity analyses revealed significant reductions in hyperconnectivity between the right middle temporal gyrus (MTG) and superior putamen, as well as between the left TPJ and left lateral prefrontal cortex (LPFC), both of which were correlated with improvements in AVH severity. These findings highlight the critical role of MTG, TPJ, PFC, and putamen in AVH pathology and suggest that rTMS may exert its therapeutic effects by modulating excessive connectivity within these circuits.

### Altered brain network properties in schizophrenia with AVH

Previous studies have established that schizophrenia is characterized by dysconnectivity across multiple large-scale networks, rather than focal abnormalities in isolated regions (28–30). Graph theory-based analyses have provided insight into the disrupted balance between segregation and integration, which may underlie core symptoms such as auditory verbal hallucinations (AVH). In the current study, schizophrenia patients at baseline exhibited impaired small-world properties, with significantly lower small-worldness (σ), clustering coefficient (Cp), local efficiency (El), and global efficiency (Eg) compared to healthy controls. These findings suggest weakened functional segregation, with compromised local clustering and inefficient processing within specialized subnetworks. The reduction in Eg further indicates impaired functional integration, reflecting disrupted brain-wide communication rather than compensatory reorganization. These alterations align with previous studies indicating that schizophrenia is associated with abnormal connectivity across distributed cortical and subcortical networks, particularly within language, sensory processing, and executive control systems (31, 32).

### Effects of rTMS on functional connectivity and AVH

A key finding of this study was that rTMS treatment significantly modulated functional connectivity in networks associated with AVH, particularly in MTG, TPJ, PFC, and putamen. These changes were most pronounced in responders, suggesting that the therapeutic effects of rTMS may be mediated by targeted network reorganization.

The MTG is a crucial hub for auditory processing, semantic integration, and language comprehension, and previous research has shown that hyperactivity in MTG contributes to the misattribution of internally generated speech as external voices in schizophrenia (33, 34). In this study, we observed that rTMS led to a significant reduction in connectivity between the right MTG and the superior putamen, and this reduction was correlated with improvements in AVH symptoms. The putamen, as part of the striato-thalamocortical loop, is involved in sensorimotor gating and auditory processing (35). Abnormal putamen activity has been reported in schizophrenia patients with persistent AVH, where excessive connectivity between the putamen and auditory/temporal regions may contribute to the inability to distinguish self-generated thoughts from external auditory input (36, 37). The observed decrease in MTG-putamen connectivity following rTMS indicates a modulation of functional interactions between auditory processing and subcortical motor circuits, which was associated with AVH symptom reduction.

Additionally, connectivity reductions between the left TPJ and left lateral PFC were significantly associated with improvements in AVH symptoms. The TPJ is critically involved in speech monitoring, self-other distinction, and auditory perception, while the lateral PFC plays a key role in cognitive control and reality monitoring (38, 39). Dysfunction in these regions may impair the ability to suppress internally generated speech, leading to the experience of AVH. The observed reduction in TPJ-LPFC connectivity post-rTMS suggests a modification in top-down control over auditory processing, which may contribute to differences in the perception of internally generated thoughts and external stimuli. Taken together, these findings suggest that rTMS may exert its therapeutic effects by selectively modulating hyperconnectivity in key AVH-related circuits, particularly the MTG-putamen and TPJ-LPFC pathways.

One potential explanation is that changes in network efficiency may influence the differentiation between self-generated and externally perceived speech, a key deficit in schizophrenia. The self-monitoring hypothesis of AVH posits that patients misattribute internally generated speech as external due to impaired functional integration between temporo-parietal, frontal, and auditory cortices. By restoring small-world properties, rTMS may enhance top-down control from prefrontal regions to auditory processing areas, allowing patients to more accurately distinguish self-generated thoughts from external stimuli. Additionally, hyperconnectivity within AVH-related circuits, particularly between the left TPJ and prefrontal regions, was significantly reduced post-rTMS, correlating with symptom improvement. This suggests that rTMS-induced network reorganization may reduce aberrant excitability in hallucination-related pathways, thereby normalizing auditory processing. These findings support the network dysregulation model of AVH, where altered small-world properties contribute to perceptual misattributions. By demonstrating that rTMS can partially restore these properties, our study provides a mechanistic framework for understanding its therapeutic effects.

Taken together, these findings suggest that rTMS exerts its therapeutic effects through targeted network reorganization, rather than solely through broad cortical inhibition. Specifically, rTMS-induced modulation of the TPJ, MTG, and associated pathways may restore functional balance in AVH circuits, leading to a reduction in hallucinatory experiences. These insights offer a more precise understanding of rTMS mechanisms, paving the way for optimized neuromodulation strategies tailored to AVH pathology.

### Differential response to rTMS: responders vs. non-responders

Comparative analyses revealed that responders exhibited significant reductions in connectivity, particularly in the MTG, TPJ, PFC, and putamen, whereas non-responders showed minimal or no functional connectivity changes following rTMS. This suggests that rTMS-induced network modulation is a key determinant of clinical improvement, and that a lack of connectivity change may underlie treatment resistance in non-responders. Notably, the absence of significant alterations in non-responders aligns with previous studies suggesting that some schizophrenia patients exhibit treatment-resistant neural circuits, potentially requiring alternative stimulation parameters or multimodal interventions to achieve therapeutic effects. Future studies should explore whether baseline connectivity patterns can predict treatment response, as this may allow for more personalized neuromodulation strategies.

### Implications and future directions

These findings provide valuable insights into the neural mechanisms underlying AVH and the therapeutic effects of rTMS. The study highlights that AVH in schizophrenia is associated with hyperconnectivity in specific cortical-subcortical circuits, particularly involving the MTG, TPJ, putamen, and PFC. The observed reductions in connectivity post-treatment suggest that rTMS may alleviate hallucinations by modulating aberrant auditory-linguistic and sensorimotor networks. However, not all patients responded to treatment, emphasizing the need for biomarker-driven approaches to identify optimal candidates for rTMS interventions. Future research should explore the relationship between functional and structural connectivity, as well as dynamic network changes over time, to refine our understanding of how rTMS influences brain function in schizophrenia.

### Limitations

Several limitations should be acknowledged. First, the sample size was relatively small, which may limit the generalizability of the findings. Second, only functional connectivity was examined, whereas structural connectivity alterations could provide additional insights into the mechanisms of AVH. Finally, the study lacked a sham control group, which precludes definitive conclusions about the specificity of rTMS effects. Future studies with larger samples, multimodal imaging approaches, and individualized stimulation protocols are needed to further optimize rTMS interventions for AVH in schizophrenia.

## Conclusions

This study demonstrated that schizophrenia patients with auditory verbal hallucinations (AVH) exhibit disrupted small-world network properties and abnormal functional connectivity, particularly in MTG, TPJ, PFC, and putamen. Low-frequency rTMS targeting the left TPJ partially led to changes in network efficiency, including increases in small-world topology, local and global efficiency, and modulation of hyperconnectivity in key cortical-subcortical circuits. These findings support the network dysregulation model of AVH and suggest that rTMS may alleviate symptoms by restoring dysfunctional connectivity patterns. However, variability in treatment response highlights the need for biomarker-driven strategies to optimize neuromodulation approaches. Future research should focus on identifying predictors of rTMS response, refining individualized stimulation protocols, and exploring synergistic interventions to enhance therapeutic efficacy in schizophrenia patients with AVH.

## Data Availability

The raw data supporting the conclusions of this article will be made available by the authors, without undue reservation.
